# Refactoring of a synthetic raspberry ketone pathway with EcoFlex

**DOI:** 10.1186/s12934-021-01604-4

**Published:** 2021-06-10

**Authors:** Simon J. Moore, Yonek B. Hleba, Sarah Bischoff, David Bell, Karen M. Polizzi, Paul S. Freemont

**Affiliations:** 1 Centre for Synthetic Biology and Innovation, Imperial College London, South Kensington Campus, Exhibition Road, London, SW7 2AZ UK; 2 Department of Life Sciences, Imperial College London, South Kensington Campus, Exhibition Road, London, SW7 2AZ UK; 3 Department Section of Structural and Synthetic Biology, Department of Infectious Disease, Imperial College London, South Kensington Campus, Exhibition Road, London, SW7 2AZ UK; 4 Department of Chemical Engineering, Imperial College London, South Kensington Campus, Exhibition Road, London, SW7 2AZ UK; 5 Present Address: School of Biosciences, University of Kent, CT2 7NJ Canterbury, England; 6 The London Biofoundry, Imperial College Translation & Innovation Hub, White City Campus, 80 Wood Lane, London, W12 0BZ UK; 7 Dementia Research Institute Care Research and Technology Centre, Imperial College London, Hammersmith Campus, Du Cane Road, London, W12 0NN UK

**Keywords:** Synthetic biology, Fine chemicals, Tyrosine, Raspberry ketone, Golden Gate, *E. coli*

## Abstract

**Background:**

A key focus of synthetic biology is to develop microbial or cell-free based biobased routes to value-added chemicals such as fragrances. Originally, we developed the EcoFlex system, a Golden Gate toolkit, to study genes/pathways flexibly using *Escherichia coli* heterologous expression. In this current work, we sought to use EcoFlex to optimise a synthetic raspberry ketone biosynthetic pathway. Raspberry ketone is a high-value (~ £20,000 kg^−1^) fine chemical farmed from raspberry (*Rubeus rubrum*) fruit.

**Results:**

By applying a synthetic biology led design-build-test-learn cycle approach, we refactor the raspberry ketone pathway from a low level of productivity (0.2 mg/L), to achieve a 65-fold (12.9 mg/L) improvement in production. We perform this optimisation at the prototype level (using microtiter plate cultures) with *E. coli* DH10β, as a routine cloning host. The use of *E. coli* DH10β facilitates the Golden Gate cloning process for the screening of combinatorial libraries. In addition, we also newly establish a novel colour-based phenotypic screen to identify productive clones quickly from solid/liquid culture.

**Conclusions:**

Our findings provide a stable raspberry ketone pathway that relies upon a natural feedstock (L-tyrosine) and uses only constitutive promoters to control gene expression. In conclusion we demonstrate the capability of EcoFlex for fine-tuning a model fine chemical pathway and provide a range of newly characterised promoter tools gene expression in *E. coli*.

**Supplementary Information:**

The online version contains supplementary material available at 10.1186/s12934-021-01604-4.

## Introduction

Synthetic biology aims to refactor synthetic pathways—in synchrony with a cell’s need to grow and divide—as it seeks to provide biosustainable routes to value-added chemicals and biopharmaceuticals. Recent examples include vanillin [1], menthol [2] and semi-synthetic drugs such as artemisinin [3] and taxol [4]. Engineering fine chemical pathways in chassis such as *Escherichia coli*, often disrupts cell homeostasis [5]. Foreign enzymes that have evolved under a different context—within thermophiles, plants, fungi, archaea—may cause unpredictable knock-on effects including: poor kinetics, requirement of cofactors/metals [6], sub-optimal gene expression and/or protein folding, while codon content and cryptic regulatory features can lead to gene expression problems. To some degree the latter can be alleviated with codon-optimisation, although this can lead to rapid translation, which also carries a penalty. In general, these imbalances drain core resources and reduce growth rates. Depending on the synthetic pathway, individual products or intermediates, can also cause toxicity [7]. In this scenario, pathway bottlenecks may require engineering, such as efflux proteins to pump out toxic products [8]. To navigate some of these issues, synthetic biology can refactor gene expression to fine tune individual enzyme levels and increase efficiency of a chosen pathway, and some extent, minimise toxic effects in harmony with host metabolism [9]. To achieve this, combinatorial DNA assembly is routinely used to control individual gene expression elements: promoters, insulators, open-reading frames (ORF), ribosome-binding sites (RBS) and terminators. Previously, we released EcoFlex, an modular cloning toolkit for flexible cloning and pathway optimisation [10]. EcoFlex is a Golden Gate based hierarchical workflow for cloning genes (Level 1) and pathways (Level 2–3) for expression in *E. coli*. In this work we sought to deploy EcoFlex to tackle a high-value fine chemical pathway. We chose a 4-(4-hydroxyphenyl)butan-2-one pathway, also referred to as raspberry ketone, as a model pathway to optimise.

Natural raspberry ketone is farmed from *Rubeus rubrum* in a number of developing countries, which amongst a range of naturally sourced products, contributes to deforestation. Approximate yields of 1 g raspberry ketone per hectare of land (~ 1000 kg of fruit) are typically obtained [11]. This presents a highly inefficient purification process, which is reflected by a high market price for naturally extracted raspberry ketone—between $10,000–20,000 kg^−1^. Microbial heterologous production of raspberry ketone has resulted in titres from 1–91 mg/L using *E. coli* [11–13]. The best strategy requires growth on rich media and a high powered T7 promoter driven gene expression. Alternatively, a semi-synthetic whole-cell approach was recently used to bypass cell toxicity and make raspberry ketone to the gram per litre scale in *E. coli* [14]. To engineer a synthetic raspberry ketone biosynthetic pathway with EcoFlex, we first considered potential factors that may limit its production in *E. coli* and the design of the pathway: supply of the substrate provision (most studies feed *p*-coumarate); limited pool of malonyl-CoA (~ 35 μM) in *E. coli* [15]; and potential bottleneck steps, especially for the type III polyketide enzyme—benzalacetone synthase (*k*_cat_ = 0.1 s^−1^) [16]. Moreover, both raspberry ketone and to a greater extent, the precursor 4-hydroxybenzalacetone (HBA) are toxic to cell growth [11]. On this basis, we selected a five-step synthetic pathway to optimise with EcoFlex. We initially screened a combinatorial promoter and RBS library. From our initial exploration three issues emerged: low success rate, low titres and loss of pathway genes through homologous recombination at the promoter and terminator regions. While we could vary the Rho-independent synthetic terminators, by selecting them from a library originally developed for resistance to homologous recombination [17], we desired further control at the promoter region. Therefore, in this work, we newly develop a degenerate σ^70^ promoter library to fine-tune enzyme levels. To overcome the initial low hit rates, we also identify a novel colour-based screening approach to identify optimal promoter design space for production of a raspberry ketone precursor, and individually tailor each enzyme level to provide maximal metabolic flux balanced with minimal promoter strength; this reduces resource usage and metabolite toxicity in the system. Overall, we provide a 65-fold improvement (up to 12.9 mg/L) in raspberry ketone production in *E. coli*. In conclusion, we report the refactoring of a synthetic raspberry ketone pathway, as an example of a synthetic biology design-build-test-learn approach to engineering a toxic fine chemical pathway in *E. coli*.

## Results and discussion

### Refactoring in *E. coli* identifies pathway bottlenecks and genetic instability

Based on available literature, we designed a synthetic raspberry ketone pathway (see material and methods), from plant, bacterial and fungal sources. The pathway begins from L-tyrosine, which is an endogenous primary metabolite, for expression in *E. coli*. The four-step pathway uses tyrosine ammonia lyase (TAL), *p*-coumaroyl-CoA ligase (PCL), benzalacetone synthase (BAS) and a NADPH-dependent raspberry ketone synthase (RKS). To complement this, we also selected a malonyl-CoA synthetase (MatB) for malonyl-CoA regeneration from malonate, CoA and ATP (17).

To begin, we built a plasmid with each gene under the control of the low-strength J23114 promoter (pJ23114-RK) and a strong RBS (PET-RBS), and tested it in a number of *E. coli* strains. We rationalised that using a weak promoter strength should provide sufficient levels of raspberry ketone to detect, allowing us to optimise the pathway by testing stronger promoters. Unexpectedly, the *recA*^*−*^ deficient *E. coli* DH10β cloning strain provided the highest yields over other common laboratory strains (Additional file 1: Figure S1). In addition, provisional experiments in different media (M9, M63, LB, 2YT and TB) confirmed that the *E. coli* DH10β pJ23114-RK plasmid strain was only productive in rich media (data not shown). Raspberry ketone production in minimal media was below the limit of detection on HPLC–MS. While minimal media is preferred for standardisation in synthetic biology, within the context of our experiment, we decided to continue with rich media (2YT) for all experiments including promoter and enzyme characterisation. Poor raspberry ketone production has also been previously reported in minimal media [11, 13]. Finally, we also observed only trace levels of raspberry ketone in either batch or in high cell density fermenter growth in the semi-defined SM6 media (Additional file 1: Figure S2), which we performed with our final optimised strain described later.

Next, we continued with *E. coli* DH10β as a heterologous host and 2YT medium supplemented with 10 mM malonate (for MatB catalysed malonyl-CoA regeneration) for screening. Then we made a selection of pathway variants to identify potential bottlenecks. Simultaneously, we attempted to assemble five pathways (*tal*-*pcl*-*matB*-*bas*-*rks*) with MoClo, each varied with one gene controlled by a strong J23100 promoter, with the remaining four genes under the control of weak strength J23114 promoter. Interestingly, after MoClo assembly of J23100-*tal* and J23114-*pcl*, J23114-*bas*, J23114-*matB* and J23114-*rks*, after *E. coli* transformation, a range of colony sizes were obtained. Despite several attempts, we were unable to isolate the desired pathway combination, suggesting this design was unstable due to overproduction of TAL. However, the remaining four pathway variants were obtained with a weak J23114 promoter for *tal expression*, while the *pcl*, *bas*, *matB* and *rks* genes were individually overexpressed with the strong J23100 promoter to increase gene expression. Interestingly, with J23100-*pcl* (and J23114-*tal*, J23114-*bas*, J23114-*matB* and J23114-*rks*), the pathway was inactive—no *p*-coumarate, HBA or raspberry ketone was detected. However, since our LC–MS analysis does not include the intracellular intermediate *p*-coumaroyl-CoA (pCA-CoA), we are unsure why this variant disrupts pathway flux. To test this further, we also built a three-gene pathway (*tal*, *pcl*, and *bas*) solely with a J23100 promoter, in an attempt to maximise HBA levels. Interestingly, this variant pathway only produced trace levels of either *p*-coumarate (~ 0.5 μM) or HBA (~ 0.5 μM), while the level of the L-Tyr substrate remained unaltered in comparison to an empty vector control. Next, for the strong J23100-*bas* variant, HBA (9 μM) levels increased 4.7-fold in comparison to the low strength promoter positive control strain (1.9 μM), confirming that BAS (expression and activity) is rate-limiting [11, 16]. At this level of HBA production, there was a significant (24.7%) decrease in the final OD_600_ of cultures grown for 48 h (*p* value = 0.02); we suspect this is due to HBA accumulation and/or gene expression toxicity from overproduction of BAS. Next, with J23100-*matB* (all other genes J23114), pathway activity remained unchanged. Finally, with J23100-*rks*, there was complete flux of HBA into raspberry ketone (2.7 μM), which confirmed that RKS strongly favours product formation. In summary, these findings suggested the pathway could be improved using EcoFlex. However, since the BAS enzyme represents a key bottleneck, we next sought to optimise this step.

### A rapid pigment screening strategy for HBA/raspberry ketone production

**Fig. 1:**
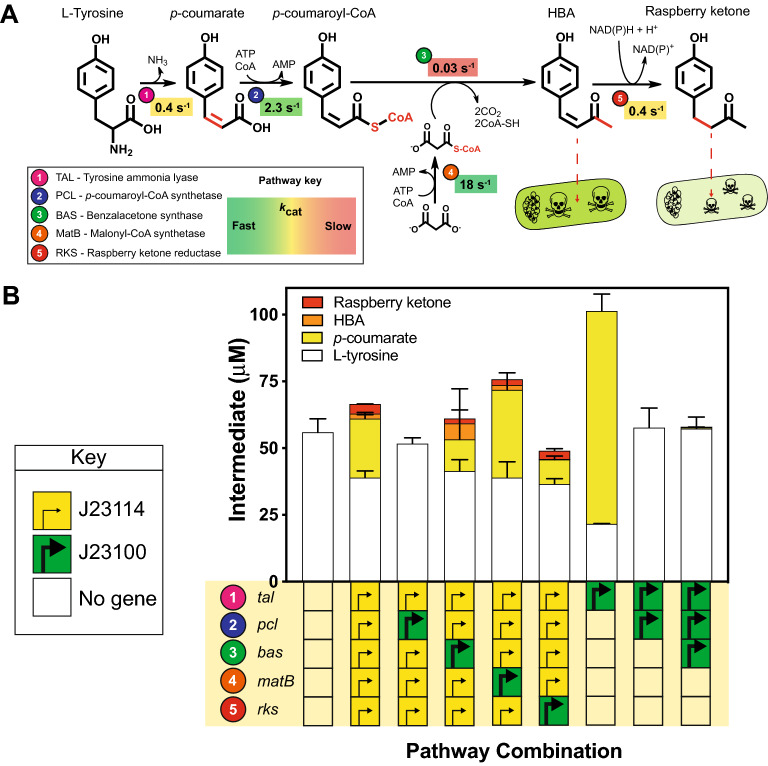
A comparison of raspberry ketone biosynthesis in vitro and in vivo identifies pathway limitations. **A** Biosynthetic pathway for raspberry ketone and *K*_cat_ values are referenced within the main text. Intermediates quantified throughout LC–MS include L-tyrosine (white box), *p*-coumarate (yellow box), HBA (orange box) and raspberry ketone (red box). **B** EcoFlex refactoring of the raspberry ketone pathway. See methods for details of growth conditions. Data and error bars (standard deviation) is representative of three biological repeats

**Fig. 2:**
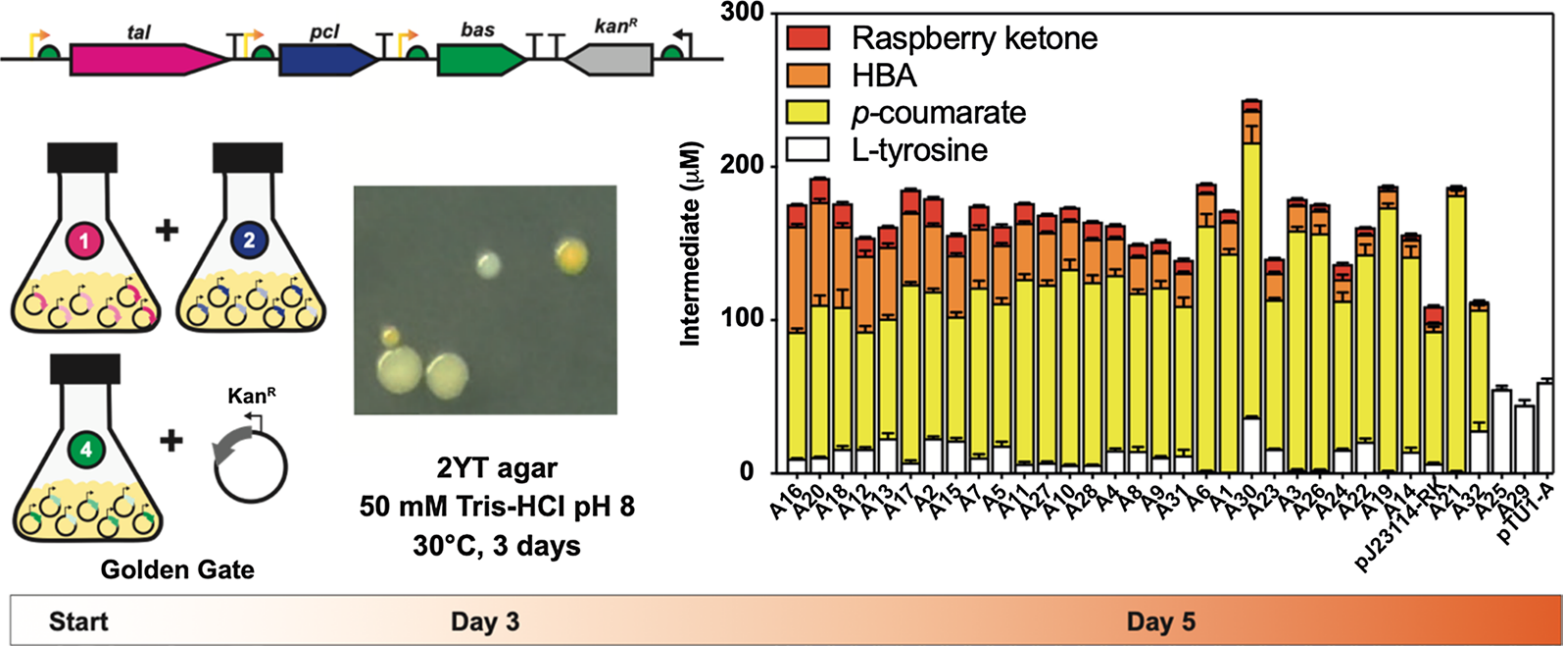
Development of colour-based phenotypic screen for HBA production. Pathway design of the HBA module with a secondary marker (kanamycin resistance) and promoter library assembly. Yellow-orange colonies that were used for selection of active pathway variants were identified after ~ 3 days of growth at 30 °C on 2YT agar supplemented with 50 mM Tris–HCl pH 8 buffer, followed by liquid growth (from triplicate single colonies) and quantification of pathway intermediates by LC–MS. Sequencing summary is provided in Additional file 1: Table S2. Data and error bars (standard deviation) is representative of three biological repeats

A key problem with fine chemical pathways is the lack of a fast, high-throughput method to optimise pathway design; raspberry ketone is colourless and requires analytical methods such as HPLC for detection. Interestingly, a retro-aldol synthesis of the precursor HBA [18], revealed this to be a yellow-orange powder, which broadly absorbs between ~ 300–450 nm in neutral-alkaline conditions (> pH 7.5) but is colourless below pH 7. Importantly, despite some absorbance between 260–350 nm, none of the other pathway intermediates such as L-Tyr (colourless), *p*-coumarate (pale yellow) or *p*-coumaroyl-CoA (pale yellow) or raspberry ketone (colourless) shares this distinct visual spectral property. Based on this observation, we tested if we could monitor HBA production using EcoFlex, by first optimising the concentrations of only the first three genes, *tal*, *pcl* and *bas* to maximise HBA accumulation. To simplify this further, we also omitted the malonyl-CoA regeneration scheme since the *E. coli* malonyl-CoA pool is ~ 35 µM [15] and the BAS enzyme has a favourable *K*_*M*_ (23.3 μM) for malonyl-CoA [16]. Therefore, at low (~ µM) levels of flux, we did not expect malonyl-CoA to be rate-limiting. Next, we initially built the pathway with a random promoter library containing ten low to high-strength EcoFlex σ^70^ promoters into a medium-copy (ColE1) origin of replication pTU2-A destination vector, while also adding a kanamycin marker as an additional selective pressure to maintain plasmid stability. After three days of incubation at 30 °C on 2YT plates, we observed a range of white-, yellow- and orange-coloured colonies (typically ~ 100–1000 per *E. coli* transformation). To continue, we picked 32 colonies that displayed strong yellow-orange pigmentation and grew in small-scale liquid culture (Figs. 1, 2). Interestingly, a number of the strains all but depleted the L-Tyr substrate (~ 1.6–2.3 μM) and had high levels of *p*-coumarate (154–180 μM), but no HBA or raspberry ketone. In addition, two strains had increased HBA levels up to 68 μM, and 14 μM raspberry ketone from an unidentified and non-specific *E. coli* alkene reductase [11]. Overall, all strains producing HBA had a weak promoter with the *pcl* gene (J23114, SJM914), while the *bas* gene had either a medium (SJM908, SJM910) or strong promoter (J23100). However, this new library also contained a number of recombination events between the *pcl* and *bas* genes, but only where the J23114 promoter was found preceding the *pcl* gene. This is probably due to its frequent occurrence within the library, as a weak strength promoter. From recent literature [19], this was due to the repetitive use homology between the promoters and the Bba_B0015 terminator in the Golden Gate assembly. Therefore, we aimed to increase the diversity of terminators used in the next round of assembly. In addition, to fine-tune individual enzyme levels, we also desired a wider selection of promoters. To achieve this, we built and characterised a new σ^70^ promoter library with degenerate bases to minimise homologous recombination.

### Characterisation of a new EcoFlex promoter library

**Fig. 3:**
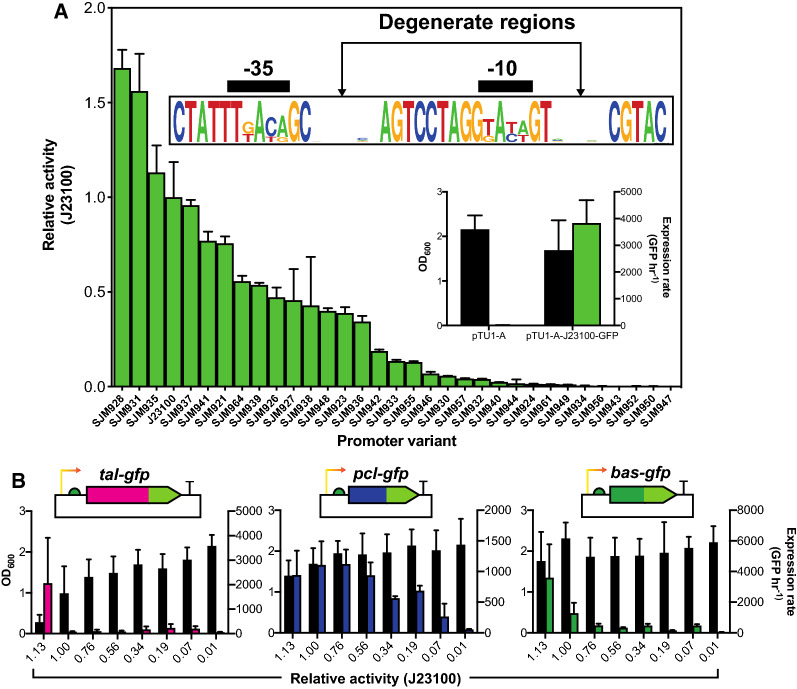
Characterisation of promoter activity with HBA module enzymes with a C-terminal GFP translational fusion.** A** Design of a degenerate σ^70^ promoter library for characterisation with eGFP in microplate conditions, for full details see methods. **B** Promoter characterisation with HBA module enzyme fusions. Black bars represent OD_600_, while coloured (pink/green/blue) bars represent eGFP fusions in terms of relative expression rate during exponential growth (GFP hr^−1^). Data is representative of two technical readings and four biological repeats grown from single *E. coli* colonies. Error bars represent standard deviation

Ultimately, our final goal was to create an optimised pathway using constitutive promoters, rather than inducible promoter control. We were intrigued by the disturbances in growth for some pathway constructs in response to stronger promoters and whether this is a common observation between different pathway enzymes. Therefore, we explored potential design faults at the level of individual gene expression for the first three pathway enzymes (TAL, PCL and BAS), which are responsible for HBA biosynthesis. To provide new variable strength σ^70^ promoters with reduced homology, we also expanded the EcoFlex promoter library [10]. This is based on the original Anderson promoter library [20] with degeneracy within the − 35 and − 10 boxes. In addition, to reduce homologous recombination, we also included two separate degenerate regions within the promoter. To assess how the new degenerate σ^70^ promoters control *E. coli* growth and the synthesis of specific proteins within the raspberry ketone pathway, we characterised eight σ^70^ promoters, spanning from low (1% activity relative to J23100) to high (SJM935—113%) activity (Additional file 1: Table S1). To monitor relative protein synthesis, we created a C-terminal eGFP translational fusion with the TAL, PCL and BAS proteins and monitored growth and fluorescence (Fig. 3B). As expected, the strongest promoter (SJM935) gave the strongest levels of eGFP fluorescence for both TAL and BAS. However, over two independent measurements, we observed strong variability in both growth and fluorescence (Fig. 3B) between the datasets for the strongest promoters (SJM935 and J23100) in contrast to more consistent growth for the low-medium strength variants. For example, some biological repeats for both SJM921-*tal*-*eGFP* and SJM921-*bas*-*eGFP* spontaneously lost GFP fluorescence during growth and demonstrated major lag times (> 2–4 h), before late exponential growth and loss of fluorescence. In addition, colonies from these clones were variable in size and smaller on average. This suggested strong promoter-gene combinations accumulate spontaneous mutations or loss of the plasmid during growth. While we did not investigate this observation in detail, we acknowledge that *E. coli* DH10β has a high background mutation rate, 13.5-fold higher than MG1655 [21]—the likely cause of genetic instability in response to toxicity. Intriguingly, these growth observations were also dependent on the gene studied. For example, for the PCL-eGFP fusions, while the final cell density decreased with increasing promoter strength for PCL variants, we did not observe any major variations in growth or fluorescence between biological repeats. This may be reflective of a specific level of toxicity to the system, which in the case of TAL, is likely due to depletion of L-tyrosine as a core metabolite. The case for the toxicity with single gene overexpression of *bas* is less clear but may be linked to differences in translation elongation/ribosome occupancy. While there was a clear trend between eGFP characterised promoter strength and fluorescence for the PCL-eGFP and BAS-eGFP fusion proteins, we were intrigued by the distinct and variable findings with TAL-eGFP (Fig. 3A). Therefore, we sought to investigate the gene-eGFP fusions separately using denaturing SDS-PAGE to assess fusion protein solubility. To do this, we selected plasmid strains with either a low, medium or high-strength promoter for the *tal-gfp*, *pcl-gfp* and *bas-gfp* fusions and analysed intracellular proteins by denaturing PAGE (Additional file 1: Figure S3). Firstly, all GFP fusions were synthesised at the expected molecular size. Under a high-strength promoter (SJM935—113%), all three fusion proteins were predominantly located in the insoluble fraction rather the soluble fraction. In particular, in comparison to TAL, the relative levels of insoluble protein for PCL and BAS decreased from the SJM935 promoter plasmids. Non-specific protein aggregation is a common issue with strong heterologous protein production in *E. coli* [22]. In contrast, both the low-strength (SJM942—18%) and medium-strength (SJM964—56%) promoter combinations gave major bands on SDS-PAGE for the soluble enzyme-GFP fusions (Additional file 1: Figure S4) and there was much less fusion protein observed in the insoluble fractions (Additional file 1: Figure S4). Therefore, as expected, the strongest promoters favour formation of inclusion bodies and drain resources quicker, resulting in decreased growth rates. In summary, these results confirmed that the characterisation of individual gene-fusions differ in relation to enzyme function and the state of protein folding within the cell. This is a form of context dependency, which is an increasingly important factor in the design of genetic circuits and pathways in synthetic biology [23]. Next, with this new knowledge, we set upon refactoring the pathway to optimise raspberry ketone production.

### Refactoring an optimised raspberry ketone pathway

**Fig. 4:**
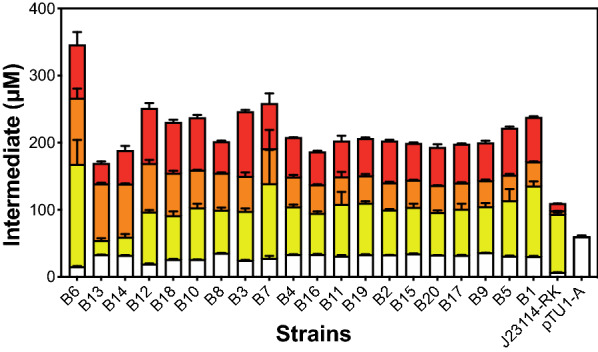
Optimisation of the HBA module with the new promoter library. Promoter fine-tuning of the HBA module. Six degenerate σ^70^ promoters were selected for EcoFlex assembly with each pathway gene, *E. coli* DH10β was transformed and grown on 2YT agar with antibiotics for 3 days at 30 °C. Colonies with distinct yellow-orange colouration were grown and quantified for pathway intermediates as described in the methods. The low-strength pathway (J23114-RK) and an empty plasmid control (pTU1-A) were tested for relative comparison. Data and error bars (standard deviation) is representative of three biological repeats. Sequencing summary is provided in Additional file 1: Table S2

To reach a balanced pathway, we next separately optimised the pathway as two modules, HBA biosynthesis and HBA reduction (raspberry ketone synthesis), before joining this together in a final assembly. We started with a focused library to fine-tune HBA synthesis (Fig. 4) applying the following rationale. Firstly, high synthesis of TAL leads to toxicity and the formation of inclusion bodies. However, since TAL has a relatively low catalytic rate and a high K_M_ for L-Tyr, we selected six medium–high strength σ^70^ promoters (SJM964, SJM926, SJM923, SJM936, SJM942, SJM933) for the *tal* gene. Secondly, PCL is a highly efficient enzyme, while too much inactivates the pathway. Therefore, six low-strength σ^70^ promoters (SJM940, SJM924, SJM956, SJM947, SJM952, SJM949) were paired with the *pcl* gene. Finally, we knew BAS was rate-limiting from all previous experiments, while high synthesis leads to formation of inclusion bodies and plasmid instability. Therefore, *bas* was paired with six medium–high strength σ^70^ (SJM928, SJM931, SJM935, J23100, SJM937, SJM941) promoters to provide a trade-off between growth and performance. The advantage of using a focused promoter strategy is that the library can be populated with only a finite range of desirable promoter strengths, whereby the fastest growing clones (e.g., those with low-strength promoters) will not dominate within libraries. The rational selection of parts also reduced the library size from 10^3^ in to 6^3^. Additionally, we also included a different strong terminator (Bba_B0015, L3S1P51, L3S2P21) to minimise homologous recombination [9] and held the RBS position constant (PET-RBS). After assembly and *E. coli* transformation of the second library, we repeated the screening process by selecting 20 colonies displaying yellow-orange pigmentation. After growth, LC–MS analysis revealed that the strains produced a wide range of *p*-coumarate levels (21–153 μM) with strong production of both HBA (36–98 μM) and raspberry ketone (31–96 μM) (Fig. 4). From sequencing, we found nine clones were duplicated within the library, but importantly, the level of the pathway intermediates between these clones were very similar. Unexpectedly, we found that clones, 6, 10, 14, and 18 contained a novel promoter sequence driving expression of the *pcl* gene that was not part of the characterised library. The promoter (SJM965, see Additional file 1) contained a single-base pair deletion and a novel randomised region between the -10 and -35 sequences. Sequencing data from the library was clean, suggesting this promoter must have been present at low levels, but was strongly selected for in our phenotypic screen. Interestingly, despite an average decrease in final OD_600_ (48 h) of 21% (range 3.7–4.7) in comparison to an empty vector control (OD_600_ = 5.03) (Additional file 1: Figure S4), the pathways in all clones were stable based on Sanger sequencing (Additional file 1: Figure S5). For example, we did not identify any homologous recombination events and there was a clear absence of mutations with the regions sequenced (e.g., promoter, RBS and terminator regions). A summary of the sequencing is provided within the Additional file 1: Figure S5 and Table S2). Satisfied with this outcome, we next looked at optimising the reductase module, which could then be added to complete the pathway.

**Fig. 5:**
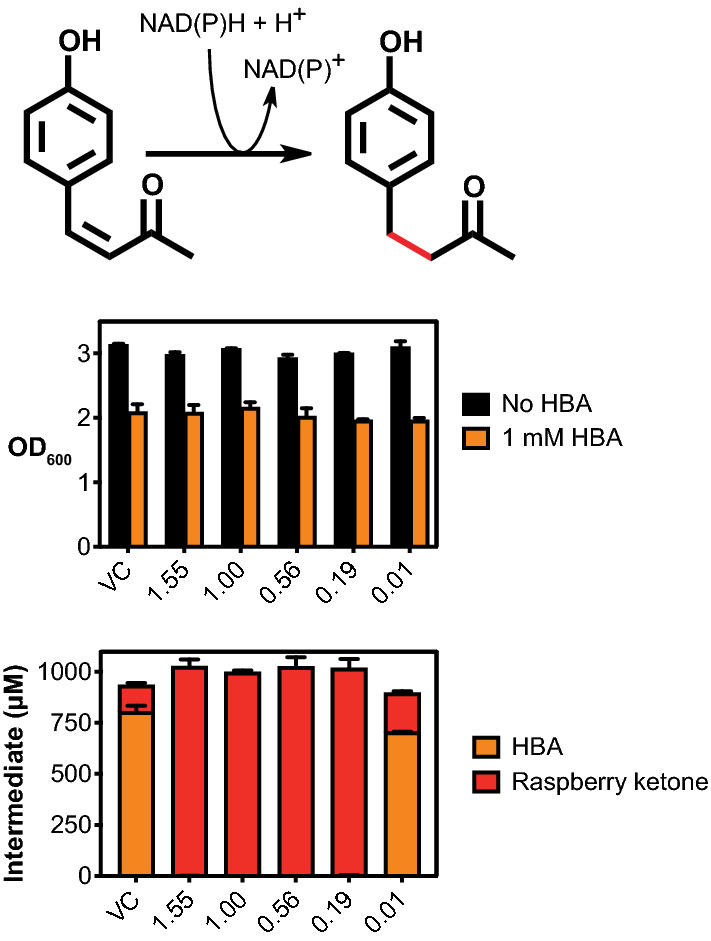
Promoter fine-tuning of the reductase module. Single promoter variants with the *rks* gene were grown in 1 mL 2YT media with antibiotics, at 30 °C, both with and without 1 mM HBA in a 12-well plate and monitored for growth (OD_600_) and LC–MS. Data and error bars (standard deviation) is representative of three biological repeats

To assess the double bond reductase, we paired the *rks* gene with five low to strong σ^70^ promoters, PET-RBS and a L2U2H09 terminator into pTU1-D-*lacZ*. The variants were grown in microtitre plates at 30 °C for 24 h and assessed for growth-rate, final OD_600_ and activity with 1 mM HBA substrate (Additional file 1: Figure S6). 1 mM HBA led to a 57% reduction in OD_600_ (Fig. 5), across the different *rks* promoter and empty-vector control strains (Fig. 5). In terms of HBA flux, four out of five of the promoter variants (high-low strength) gave 99.9% conversion of HBA into raspberry ketone (Fig. 5), with only the lowest strength promoter (SJM961—1% activity relative to J23100) leading to low conversion into raspberry ketone: 21.3 vs 13.9% with an empty vector control. Interestingly, the final OD_600_ was stable with increasing the RKS promoter strength (Fig. 5).

**Fig. 6:**
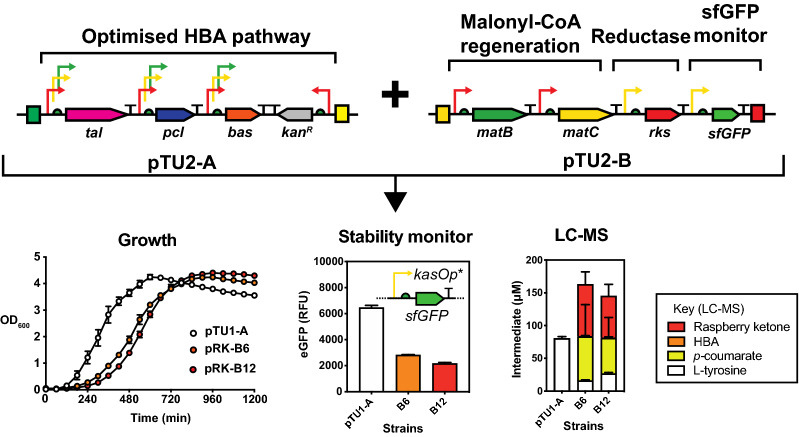
Level 3 assembly of the final optimised strain with a malonyl-CoA regeneration module, optimised reductase step and a pathway stability monitor. The plasmid strains were assessed for growth and eGFP fluorescence in 96-well microtitre plates, as well as LC–MS for pathway intermediates (representative of six biological repeats). Error bars represent standard deviation. The plasmid strains (pRK-B6 and pRK-B12) correspond to the B6 and B12 HBA modules, shown in Fig. 4 and Additional file 1: Table S5. The control plasmid (pTU1-A) is pTU1-D-kasOp*-sfGFP. Further details are described in methods

Next, we formed the final pathway design by combining two of the strongest HBA modules (B6 and B12 from Fig. 4), the optimised reductase module (from Fig. 5) and a separate malonyl-CoA regeneration module (Fig. 6). For the latter module, we aimed to increase the supply of malonate through heterologous expression of the *Rhizobium trifolii matC* (malonate transporter) gene in combination with *matB* (described earlier) under a low strength promoter in the final plasmid design. This strategy has previously been employed successfully for other flavonoid natural products [24, 25]. Additionally, we also introduced a sfGFP fluorescence reporter to monitor relative pathway stability, to act as an indicator of genetic stability [5]. To measure pathway stability and the drain of the plasmid system on general protein synthesis, we used a superfolder GFP (sfGFP) reporter, under the control of kasOp* promoter; a *Streptomyces* σ^70^ promoter that is strongly active in *E. coli* at low levels. Next, this final plasmid strain was assessed for growth and fluorescence to monitor for growth and plasmid stability (Fig. 6). The relative fluorescence of the strains was very similar between individual repeats, which suggested the strains were stable. However, relative fluorescence was reduced by 70% (Fig. 6) in comparison to the vector control (pTU1-A-kasOp*-*sfGFP*), which suggested that regardless of pathway productivity, the extra genes within the plasmid design (eight in total) cause a major drain on net protein synthesis. This was reflected by a decrease in the sfGFP reporter fluorescence and a delay of 4 h for lag growth time. Finally, we characterised the strain for relative protein levels (Fig. 6) and raspberry ketone production. SDS-PAGE revealed TAL accounts for ~ 10–20% of all intracellular proteins (Additional file 1: Figure S7), while BAS and RKS were also clearly overproduced (Additional file 1: Figure S7). At the metabolite level, we observed a clear 67–81% decrease in the L-tyrosine substrate relative to the control, 54–67 μM for *p*-coumaric acid, ~ 1 μM HBA and 63–78 μM (10.8–12.9 mg/L) of raspberry ketone, respectively, in small-scale (5 mL 2YT) batch conditions. Additionally, we also did separate experiments to test the effect of malonate on malonyl-CoA regeneration, but we found this was not significantly limiting for raspberry ketone production in *E. coli* (Additional file 1: Figure S8). This might be due to the kinetic properties of the BAS enzyme, which has a low *K*_*M*_ (23.3 μM) for malonyl-CoA [16] and is favourable for *E. coli* to replace under homeostasis. Overall, from starting with an initial design that produced barely trace levels of raspberry ketone (0.2 mg/L), we have achieved a maximum 65-fold (12.9 mg/L) improvement in titre and have produced a stable pathway using only constitutive expression.

## Conclusions

Currently, an array of fine chemicals and medicinal compounds require chemical synthesis or extraction from the natural source, which can be costly, inefficient or require extensive agricultural land—this is especially important as we transition to a sustainable bioeconomy. A key aim for synthetic biology within the next decade or so [26] is the replacement of these strategies using efficiently engineered microbial or plant chassis. Our work demonstrates a strategy to reduce the pressures of toxicity frequently encountered in routine DNA assembly workflow: here we used our EcoFlex MoClo platform. This is a frequent but understudied issue [5, 27] with handling toxic fine chemical pathways, and of broad interest to synthetic biology. Our EcoFlex library provides stable pathway expression for the product pathway, raspberry ketone using only constitutive control of individual pathway genes; this is more favourable than inducible promoters for industrial application [28]. However, further work is required to establish production under minimal medium conditions to exploit potential bioconversion of renewable feedstocks for value-added production of natural raspberry ketone. This is potentially achievable since the *E. coli* L-tyrosine pool can be engineered to the g/L scale [29]—a key requirement for industrial application. In addition, detoxication strategies may also be required such as the use of self-resistance mechanisms [8] or direct chemical processing via continuous extraction methods [30]. In conclusion, we provide an upgrade to our original EcoFlex MoClo system for the rapid optimisation of a toxic fine chemical in *E. coli*, by refactoring pathway expression to the minimum level for maximum pathway flux and minimal resource drainage.

## Materials and methods

### Molecular biology and general methods

All standard molecular biology, routine growth conditions and antibiotic concentrations are described in a previous publication (EcoFlex). All primers used are listed in the Table S3. For preparation of promoter libraries, 50 ng of each promoter part was included in a Level 1 MoClo reaction. 25 µL of commercial chemical competent DH10β (NEB) cells were transformed with one-third (5 µL) of the reaction mixture, using routine heat shock method and then recovered for 1 h with 200 µL of SOC medium. 50 µL of this mixture was directly incubated in 10 mL LB with ampicillin (10 μg mL^−1^) and grown for 16 h at 30 °C. Plasmid DNA was purified and sequenced (SourceBioscience).

### σ^70^ degenerate library

Forward primer Sigma70_11N_R and reverse primer Sigma70_F (see Additional File 1: Table S4) were used to amplify the library from the template pSB1C3-J23100-eGFP, as described in our previous publication [10].

### HBA synthesis

A 20% (w/v) solution of NaOH (2.00 g NaOH in 10 mL ddH_2_O) was cooled in an ice water bath. Base solution was added dropwise into 4-hydroxybenzaldehyde (2.00 g, 16.38 mmol) in acetone (12 mL) over 5 min. The resulting yellow solution was stirred in the ice bath for a further 10 min, and then at room temperature for 16 h. The reaction was quenched with 50 mL of ice-water mixture and cooled in an ice water bath. Concentrated HCl (15 mL) was added to adjust the pH to 1. The mixture was concentrated *in vacuo* and extracted with ethyl acetate (2 × 100 mL). The ethyl acetate extracts were washed with ddH_2_O (1 × 50 mL), brine (1 × 75 mL) and dried with Na_2_SO_4_. Normal phase purification (ethyl acetate: hexane gradient 2–40%), provided a yellow crystalline solid (2.02 g, 76% yield). HBA was synthesised using a published method [18] and NMR spectra (Additional file 1: Figure S6) agrees with published literature.

### HBA screening and analysis

For the HBA pathway screening, the transformation mixtures were grown for 48–72 h at 30 °C  on modified 2YT agar (Melford) until pigmentation was observed. Modified 2YT liquid medium was adjusted to pH 8 with 50 mM Tris base and HCl, before addition of 15 g L^−1^ of technical agar (Melford) to enhance colour detection. A reduced concentration of chloramphenicol (20 µg mL^−1^) and kanamycin (20 μg mL^−1^) was used for pathway screening and all liquid cultures. For growth, fluorescence and LC–MS measurements, standard 2YT medium was used. For LC–MS analysis, three single colonies (from *E. coli* transformations) were grown in in 5 mL 2YT for biological repeats. Samples for HPLC–MS were measured twice to ensure MS signals were stable over the measurement time period.

### Gene synthesis

*Rhodotorula glutinis tal*, *Arabidopsis thaliana pcl*, *Rubeus rubrum bas*, *Rhodopseudomonas palustris matB*, *Rhizobium trifolii matC* and *Rheum rubrum rks* were selected for gene synthesis by Life technologies, Invitrogen. The genes were codon optimised for *E. coli* K12 expression and compatibility with EcoFlex. Sequences provided in Additional file 1.

### Denaturing PAGE of intracellular soluble and insoluble proteins

DH10β plasmid strains were grown for 24 h at 30 °C. The OD_600_ was measured and 1 mL of culture was centrifuged. The cell pellet was then re-suspended in Buffer A with 1 mg mL^−1^ lysozyme, 1% Triton X-100 and 50 units/µL Benzonase® (Millipore) so the cells were concentrated to an equivalent OD_600_ of 20 and incubated at 30 °C for 30 min. Lysed cells were then clarified at 18,000 × *g*, 4 °C for 10 min to separate soluble and insoluble protein fractions. 10–50 μg of total protein was then separated by denaturing PAGE and stained with InstantBlue (Generon).

### Plate reader measurements

For CLARIOStar**©** (BMG Labtech) plate reader assays, a 100 µL 2YT culture volume was grown in quadruplicate biological repeats (e.g. four single colonies) as an overnight pre-culture for 16 h. The OD_600_ was recorded, before dilution into a fresh 96-well plate with 100 µL 2YT and antibiotics at a starting OD_600_ of 0.02. Cell density was measured in a plate reader at 600 nm in a 96-well Greiner plate to a starting OD_600_ of 0.02. Plates were sealed with a Breathe-Easy® membrane (Sigma) and grown at 30 °C for 6–12 h at 600 rpm. OD_600_ and GFP measurements were recorded every 10 min. The experiment was repeated on two independent days to ensure reproducibility. Error bars represent standard deviation.

### LC–MS of raspberry ketone pathway intermediates

For cultures, cells were removed by centrifugation at 6,000 × *g*, 4 °C for 10 min. The supernatant was acidified by addition of 2% (v/v) HCl, followed by clarification for 18,000 × *g*, 4 °C for 10 min. For enzyme reactions, 50 µL samples were removed and inactivated with 2% (v/v) HCl and centrifuged at 13,000 rpm for 10 min at room temperature. Samples were diluted with 450 µL ddH_2_O. Samples were then directly analysed by LC–MS, performed with an Agilent 1290 Infinity system with an online diode array detector in combination with a Bruker 6500 quadruple time-of-flight (Q-ToF) mass spectrometer. An Agilent Extend-C18 2.1 × 50 mm (1.8 μm particle size) column was used at a temperature of 25 °C with a buffer flow rate of 0.2 mL^−1^ min^−1^. LC was performed with a linear gradient of buffer A (0.1% formic acid) and buffer B (0.1% formic acid in acetonitrile). Separation was achieved using 5% buffer B for 2 min, followed by a linear gradient to 50% buffer B from 2–9 min, which was held at 50% buffer B from 9–10 min. Spectra were recorded between a mass range of 90–1000 m*/z* at a rate of 3 spectra per second. Standards were prepared and calibration curves for the intermediates L-Tyr, *p*-coumaric acid, HBA and raspberry ketone were derived (Additional File 2). Quantitation was based on the MS peak area of precursor or fragment ion in comparison to the analytical standards. Under the conditions used, raspberry ketone is detected as a sodium adduct [M + Na^+^]^+^ or as a diagnostic fragment ion at *m/z* = 107.49, corresponding to C_7_H_7_O. For the standards in solvent, good linearity (R^2^ > 0.99) was achieved over the range of 0.3 to 30 pmol on column. The lower limit of quantitation was set at 0.3 pmol. Samples that were below this limit were repeated by increasing the injection volume to 1 µL. Error bars represent standard deviation from three independent biological samples.

## Supplementary Information

**Additional file 1.** Additional figures and tables.

**Additional file 2.** Extracted ion chromatograms and example LC-MS calibration data.

## Data Availability

The majority of the data generated and analysed is included in the article. Any further details or requests regarding plasmids or datasets used and analysed are available from the corresponding author on reasonable request.
