# Correction to: Refactoring of a synthetic raspberry ketone pathway with EcoFlex

**DOI:** 10.1186/s12934-021-01632-0

**Published:** 2021-08-18

**Authors:** Simon J. Moore, Yonek B. Hleba, Sarah Bischoff, David Bell, Karen M. Polizzi, Paul S. Freemont

**Affiliations:** 1 Centre for Synthetic Biology and Innovation, Imperial College London, South Kensington Campus, Exhibition Road, London, SW7 2AZ UK; 2 Department of Life Sciences, Imperial College London, South Kensington Campus, Exhibition Road, London, SW7 2AZ UK; 3 Department Section of Structural and Synthetic Biology, Department of Infectious Disease, Imperial College London, South Kensington Campus, Exhibition Road, London, SW7 2AZ UK; 4 Department of Chemical Engineering, Imperial College London, South Kensington Campus, Exhibition Road, London, SW7 2AZ UK; 5 Present Address: School of Biosciences, University of Kent, CT2 7NJ Canterbury, England; 6 The London Biofoundry, Imperial College Translation & Innovation Hub, White City Campus, 80 Wood Lane, London, W12 0BZ UK; 7 Dementia Research Institute Care Research and Technology Centre, Imperial College London, Hammersmith Campus, Du Cane Road, London, W12 0NN UK

## Correction to: Microb Cell Fact (2021) 20:116 https://doi.org/10.1186/s12934-021–01604-4

Following publication of the original article [1], the authors identified an error in Fig. 1b.

The correct Fig. 1b and its caption is given in this erratum. The original article has been revised.

**Fig. 1:**
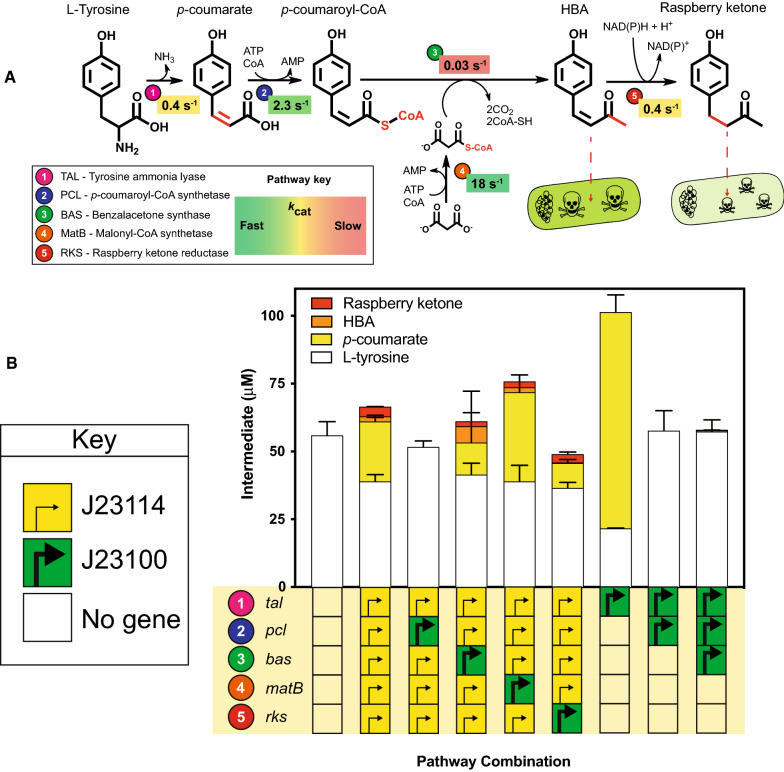
A comparison of raspberry ketone biosynthesis in vitro and in vivo identifies pathway limitations. **A** Biosynthetic pathway for raspberry ketone and *K*_cat_ values are referenced within the main text. Intermediates quantified throughout LC–MS include L-tyrosine (white box), *p*-coumarate (yellow box), HBA (orange box) and raspberry ketone (red box). **B** EcoFlex refactoring of the raspberry ketone pathway. See methods for details of growth conditions. Data and error bars (standard deviation) is representative of three biological repeats

## References

[1] Moore SJ, Hleba YB, Bischof S, Bell D, Polizzi KM, Freemont PS (2021). Refactoring of a synthetic raspberry ketone pathway with EcoFlex. Microb Cell Fact..

