# Supplementary material for: Refactoring of a synthetic raspberry ketone pathway with EcoFlex
Source: Microb Cell Fact. 2021 Jun 10;20:116. doi: 10.1186/s12934-021-01604-4 (PMC8193874; doi:10.1186/s12934-021-01604-4)
Supplement: Supplementary file 1 — Additional file 1. Additional figures and tables. [file 12934_2021_1604_MOESM1_ESM.docx]

**Supplementary information**

**
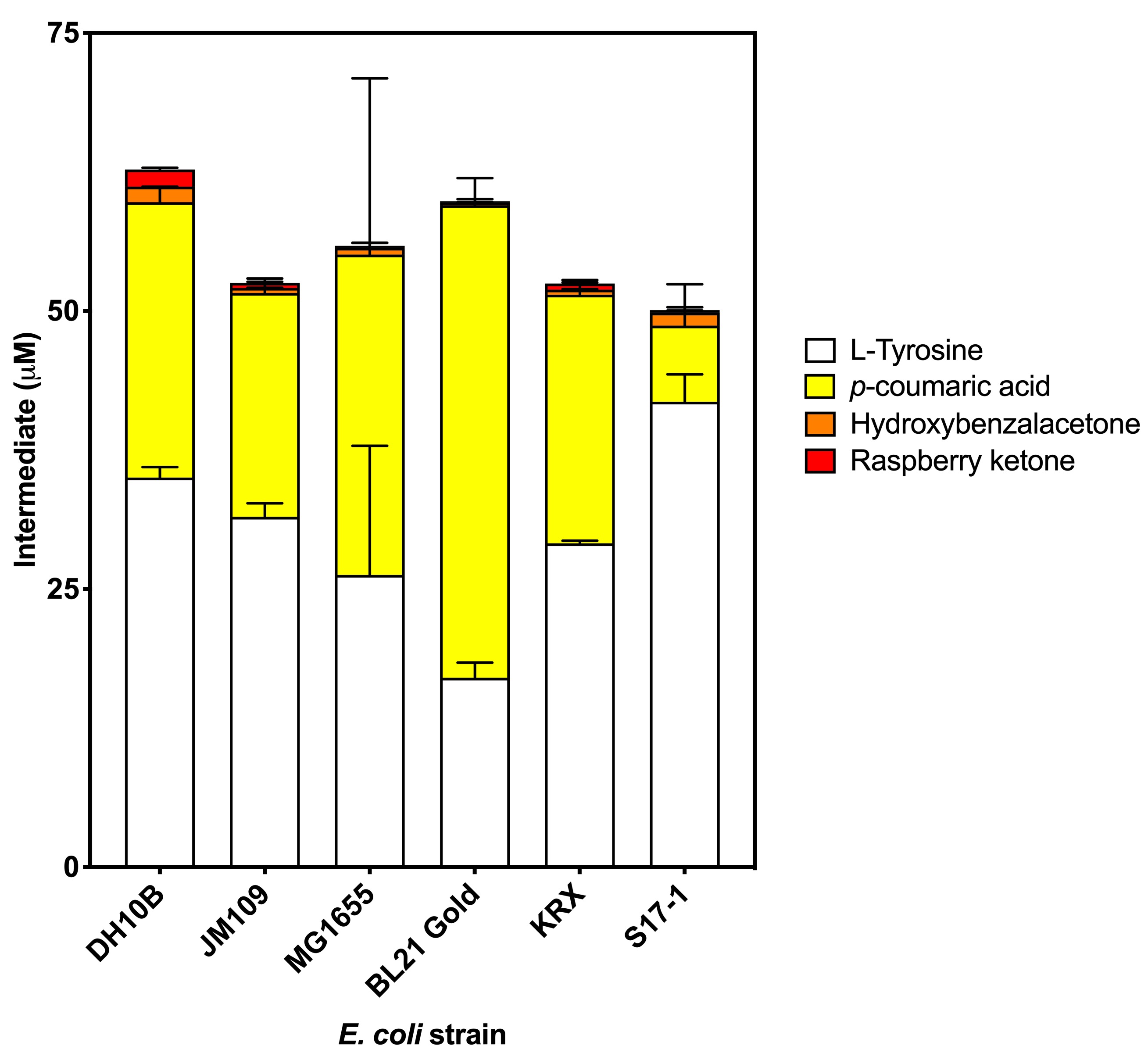
**

**Figure S1.** *E. coli* MG1655, DH10B, JM109, BL21 Gold (DE3), KRX (DE3) and S17-1 were transformed with pJ23114-RK. Cultures were grown at 30°C for 48 hours with 5 mL of 2YT medium with chloramphenicol and grown in a 12-well plate as a triplicate biological repeat. Culture supernatants were analysed by LC-MS as described within the materials and methods.

**Figure S2.** Fermenter growth of the pRK-B12 strain in SM6 minimal media (A) OD_600_ measurement. (B) LC-MS of pathway intermediates. Data is representative of two biological repeats. Fermenter conditions (Guerrero Montero et al., 2019): Cultures were grown in 1L of SM6 media at 30°C for 89 hours in a Minifors 2 (Infors HT), with 1000 rpm stirring and constant air aeration. An overnight seed culture was diluted into 1 L of fresh SM6 media with antibiotics to a starting OD_600_ of 0.5.SM6 media composition: 31.1 g/L glycerol , 5.2 g/L (NH_4_)_2_SO_4_, 4.2 g/L NaH_2_PO_4_.2H_2_O, 4.025 g/L KCl, 1.05 g/L MgSO_4_.7H_2_O, 5.2 g/L citric acid, 0.052 g/L CaCl_2_.2H_2_O, 0.02 g/L ZnSO_4_.7H_2_O, 0.0275 g/L MnSO4.4H_2_O, 0.0075 g/L CuSO_4_.5H_2_O, 0.004 g/L CoSO_4_.7H_2_O, 0.1 g/L FeCl_3_.6H_2_O, 0.0003 g/L H_3_BO_3_, 0.0003 g/L Na_2_MoO_4_.2H_2_O.

**Figure S3.** Final OD_600_ measurement of B library represented in Figure 4.

**Figure S4.** SDS-PAGE of intracellular proteins produced from different gene-eGFP fusions expressed from different promoter strengths (high - green, medium - orange and low - red). Soluble and insoluble fractions. The expected sizes of the fusion proteins are as follows: TAL-eGFP – 103 kDa; PCL-GFP – 89 kDa; and BAS-GFP – 70 kDa. Coloured arrows indicate overproduced fusion protein. See methods for full details.

**
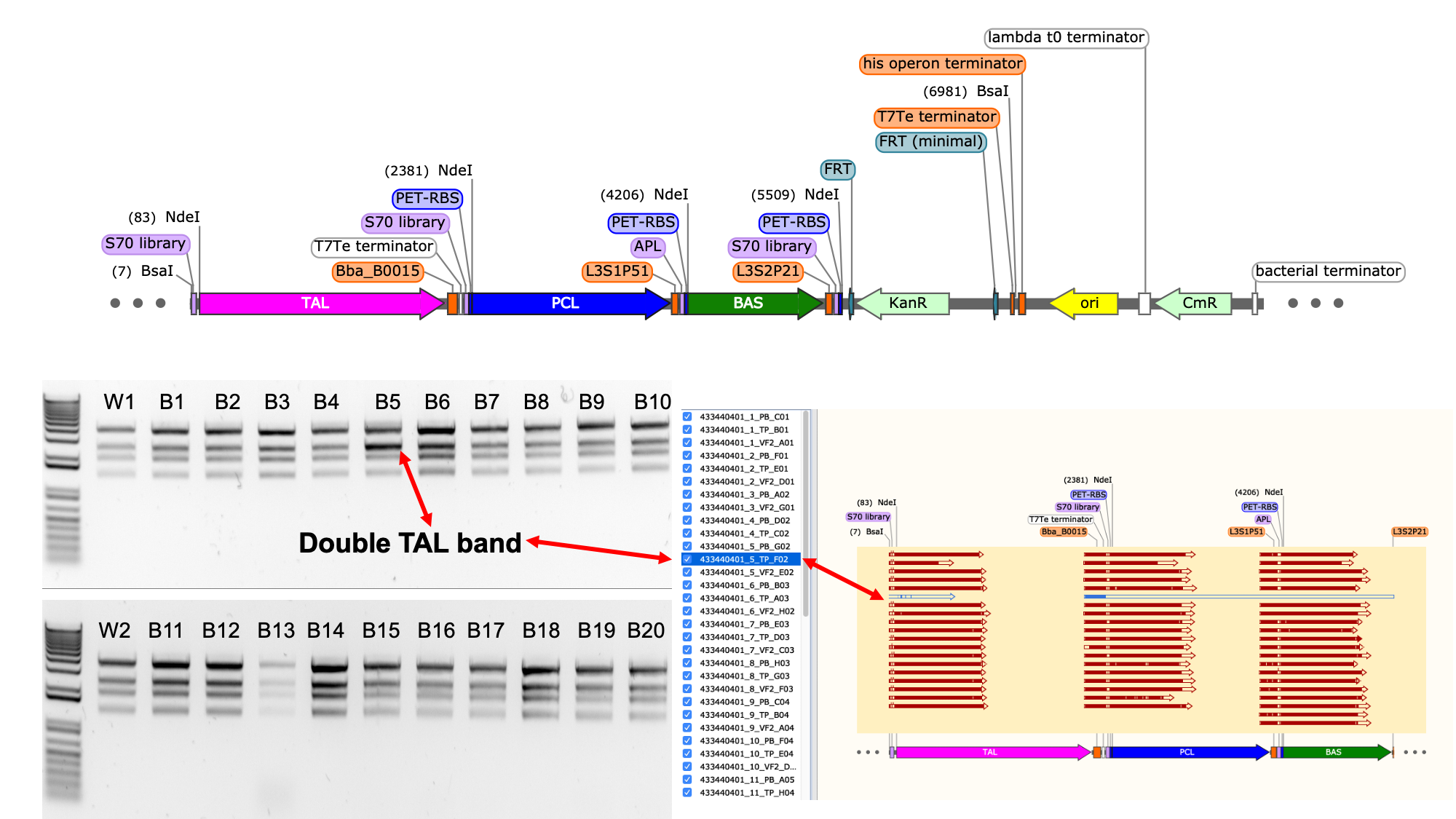
**

**Figure S5.** Plasmid design, restriction digest and DNA sequencing alignment summary (SnapGene 4.2) of the stabilised HBA module plasmid library for B1-B20 clones (selected orange colonies) and two non-productive variants W1-W2 (white colonies). Red arrows demonstrate a duplication error derived from MoClo assembly, which could not be sequenced with the standard primers (VF2, TP and PB) due two internal priming sites from a duplication of the *tal* gene.

**
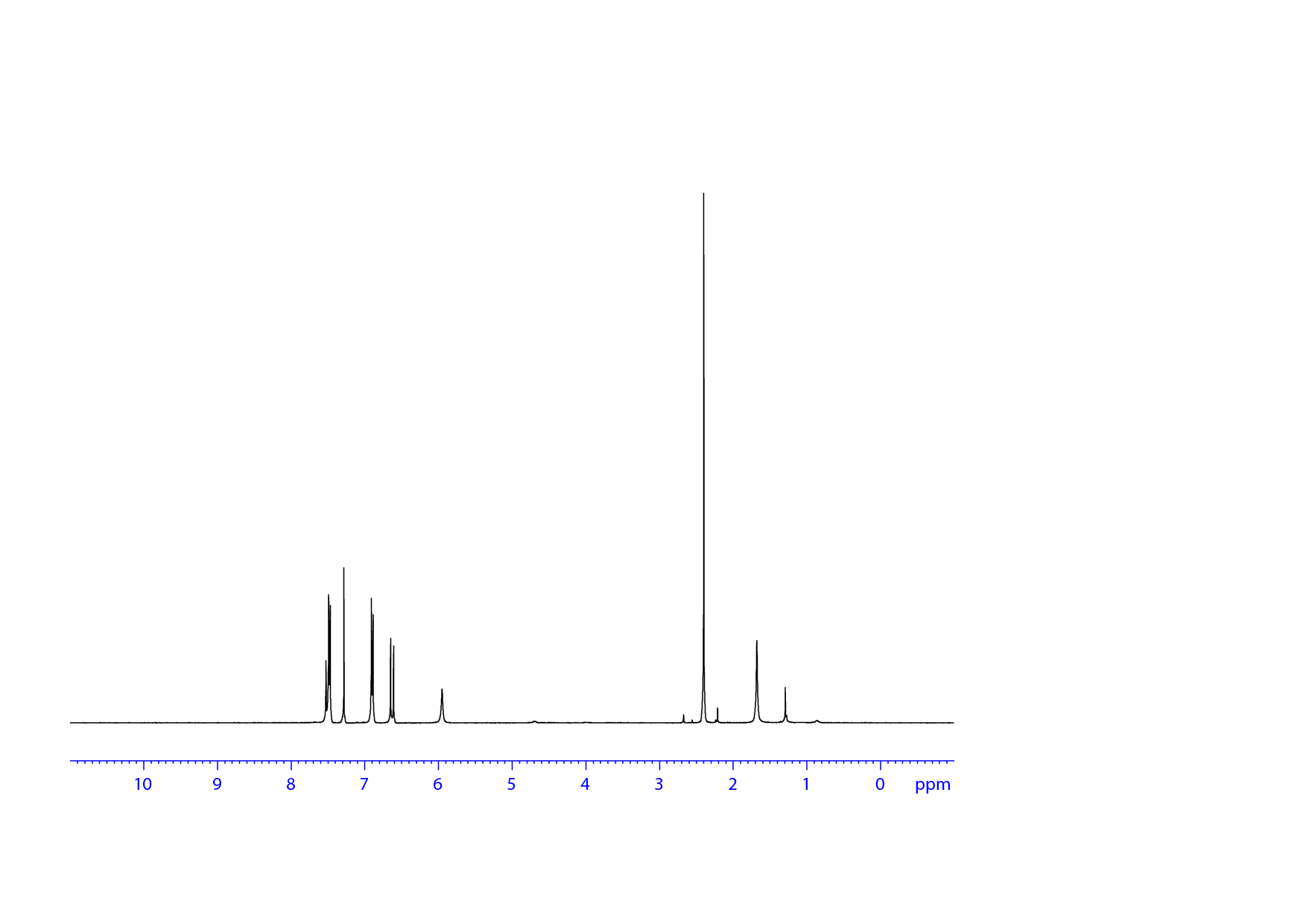
**

**Figure S6.** 400 MHz ^1^H NMR spectra of chemically synthesised HBA in CDCl_3_

**
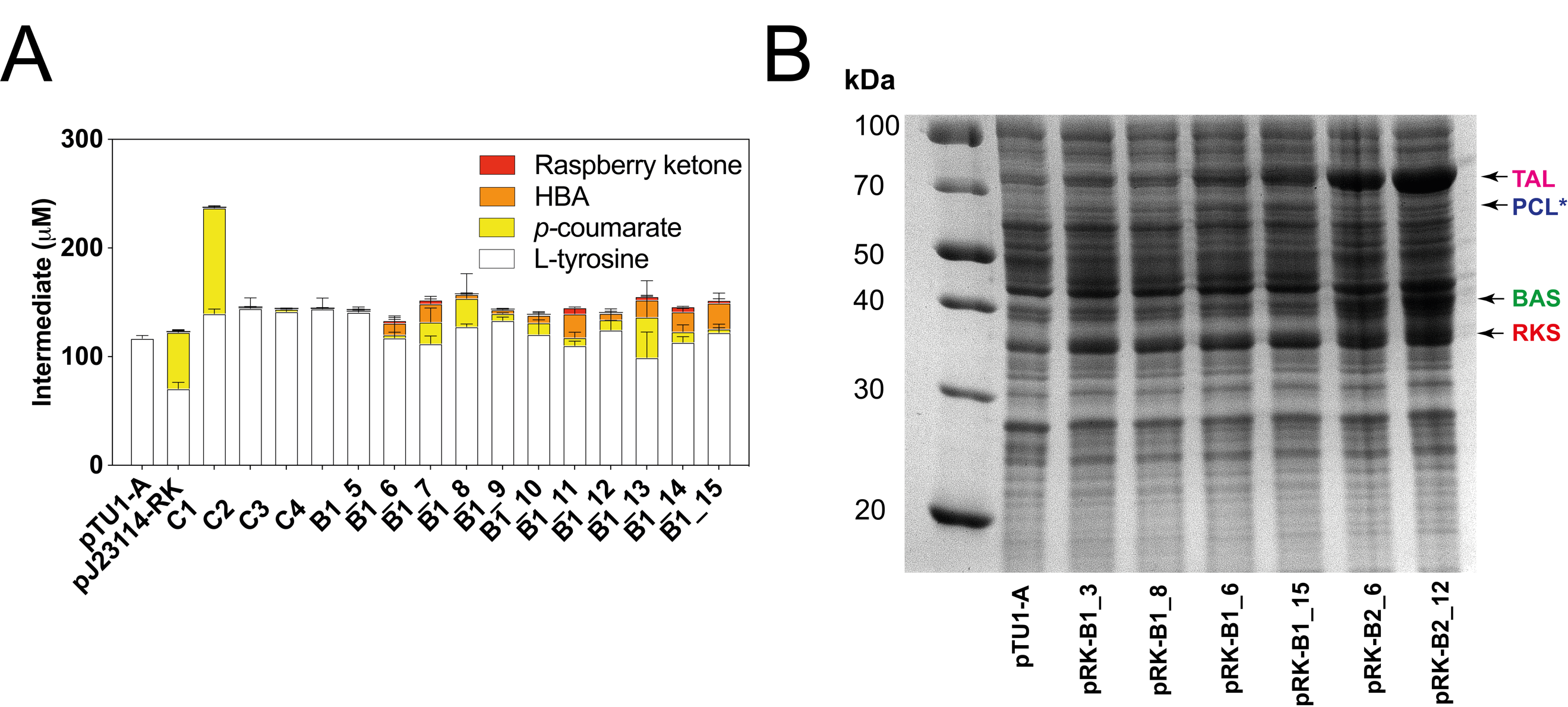
Figure S7.** Characterisation of the B libraries. (A) LC-MS data of additional B library (B1 - not presented in main manuscript), data is representative of three biological repeats. Strains for this library were assessed for protein production in comparison to pRK-B6 (same as B2_B6) and pRK-B12 (same as B2_B12). (B) SDS-PAGE of intracellular proteins from strains selected for pathway assembly (in Figure 4) from B1 and B2 libraries, representing low (B1_3, B1_8), medium (B1_8 and B1_15) and high raspberry ketone production (B2_6 and B2_12). B1 library was prepared with a random combination of the degenerate σ^70^ promoters.

**Figure S8. The effect of 10 mM malonate on raspberry ketone production.** *E. coli* DH10β pRK-B12 strain was grown in 10 mL 2YT with/without 10 mM malonate supplementation. The culture was grown as a triplicate biological repeat (see methods).

**Table S1.** Table of promoter sequences and activities

| **Promoter** | **Primer design**  CTATTTKAYRGCNNNNNNAGTCCTAGGKAYWGTNNNNNCGTAC | **Normalised**  **Activity**  **(J23100)** | **SD** |
| --- | --- | --- | --- |
| SJM928 | CTATTTGACAGCTACAGAAGTCCTAGGTACAGTGCCTGCGTAC | 1.681 | 0.096 |
| SJM931 | CTATTTGACAGCAAAGAGAGTCCTAGGTATTGTCATCTCGTAC | 1.560 | 0.198 |
| SJM935 | CTATTTGACAGCCGGAAAAGTCCTAGGTATTGTCTAGACGTAC | 1.131 | 0.143 |
| J23100 | CTATTTGACGGCTAGCTCAGTCCTAGGTACAGTGCTAGCGTAC | 1.000 | 0.185 |
| SJM937 | CTATTTGACAGCTTAACTAGTCCTAGGTACTGTAAAGGCGTAC | 0.958 | 0.028 |
| SJM941 | CTATTTGACGGCAACTCTAGTCCTAGGTATAGTGGCAGCGTAC | 0.769 | 0.049 |
| SJM921 | CTATTTTACAGCGACTCCAGTCCTAGGTATAGTAAGAACGTAC | 0.756 | 0.036 |
| SJM964 | CTATTTTACAGCGTCTACAGTCCTAGGTATAGTAGCCTCGTAC | 0.556 | 0.029 |
| SJM939 | CTATTTGACAGCCCCCCCAGTCCTAGGTATTGTGCCGTCGTAC | 0.536 | 0.012 |
| SJM926 | CTATTTGACGGCCAAGCAAGTCCTAGGTACAGTCTGGTCGTAC | 0.472 | 0.051 |
| SJM927 | CTATTTTACAGCCCCTCAAGTCCTAGGTATAGTGACCGCGTAC | 0.456 | 0.165 |
| SJM938 | CTATTTTACAGCTTACTCAGTCCTAGGTATAGTGCAAACGTAC | 0.429 | 0.256 |
| SJM948 | CTATTTGATAGCCGCCGTAGTCCTAGGTATAGTAGTAACGTAC | 0.399 | 0.015 |
| SJM923 | CTATTTGACAGCACCCCGAGTCCTAGGTACAGTCCTGGCGTAC | 0.388 | 0.031 |
| SJM936 | CTATTTGACGGCCATTCAAGTCCTAGGTACAGTCAATGCGTAC | 0.344 | 0.030 |
| SJM933 | CTATTTTACGGCTCAAGCAGTCCTAGGTATAGTAGCAACGTAC | 0.135 | 0.008 |
| SJM955 | CTATTTGACAGCTTCTCTAGTCCTAGGGATAGTACGTACGTAC | 0.130 | 0.005 |
| SJM946 | CTATTTTACAGCTAGACGAGTCCTAGGTATTGTTTCATCGTAC | 0.069 | 0.009 |
| SJM930 | CTATTTTATGGCATTATGAGTCCTAGGGACAGTATAATCGTAC | 0.057 | 0.000 |
| SJM957 | CTATTTGACAGCATCTAAAGTCCTAGGTATAGTATATGCGTAC | 0.042 | 0.003 |
| SJM932 | CTATTTTACAGCCCGAAGAGTCCTAGGTATTGTACGGACGTAC | 0.040 | 0.003 |
| SJM940 | CTATTTTACAGCTCATGAAGTCCTAGGTACAGTGCGATCGTAC | 0.024 | 0.001 |
| SJM944 | CTATTTGACAGCACGCCTAGTCCTAGGGACTGTCCGACCGTAC | 0.017 | 0.020 |
| SJM924 | CTATTTTACAGCATGGCTAGTCCTAGGGACTGTATAATCGTAC | 0.014 | 0.002 |
| SJM961 | CTATTTGACAGCCTGCCCAGTCCTAGGGACAGTACTGACGTAC | 0.013 | 0.001 |
| SJM949 | CTATTTGACAGCTCGCCGAGTCCTAGGGACAGTACTACCGTAC | 0.011 | 0.001 |
| SJM934 | CTATTTGATAGCAGGTATAGTCCTAGGTACTGTCAACTCGTAC | 0.004 | 0.002 |
| SJM956 | CTATTTTACAGCGGTCCCAGTCCTAGGGACAGTAGTACCGTAC | 0.004 | 0.001 |
| SJM943 | CTATTTGATAGCTGCCTCAGTCCTAGGTATAGTTCAGACGTAC | 0.003 | 0.000 |
| SJM952 | CTATTTGATAGCCGTACAAGTCCTAGGGATTGTCGCTTCGTAC | 0.003 | 0.000 |
| SJM950 | CTATTTTACAGCCCGCCGAGTCCTAGGGATTGTCTCACCGTAC | 0.003 | 0.001 |
| SJM947 | CTATTTGATAGCTCCCACAGTCCTAGGTATAGTCCCACCGTAC | 0.003 | 0.000 |
| SJM965 | CTATTTGACGGCCCGGACATCCTAGGTACTGTACGTCCGTAC | ND | ND |

**Table S2.** Plasmid variants and summary of metabolites detected on LC-MS

**Table S3.** Plasmids developed during this study

| ID | Description | Source / Function | Cloning method |
| --- | --- | --- | --- |
| pSJM471 | pBP-ORF-*tal* | *R. glutinis -* Synthetic gene | NdeI and  BamHI (DNA synthesis) |
| pSJM472 | pBP-ORF-*pcl* | *A. thaliana -* Synthetic gene |  |
| pSJM473 | pBP-ORF-*bas* | *R. palmatum -* Synthetic gene |  |
| pSJM475 | pBP-ORF-*matB* | *R. palustris -* Synthetic gene |  |
| pSJM476 | pBP-ORF-*rks* | *R. idaeus -* Synthetic gene |  |
| pSJM478 | pBP-ORF-*kan^R^*-FRT | pKD13 |  |
| pSJM599 | pBP-ORF-*matC* | *R. trifoli -* Synthetic gene | NdeI and BamHI (PCR) |
| pSJM867 | pBP-ORF-*pcl-*NoStop | Same as pSJM330 with TAA stop codon removed | NdeI and BamHI (PCR) |
| pSJM868 | pBP-ORF-*bas-*NoStop | Same as pSJM331 with TAA stop codon removed | BsaI (PCR) |
| pSJM882 | pBP-ORF-*tal-*NoStop | Same as pSJM329 with TAA stop codon removed | BsaI (PCR) |
| pSJM876 | pBP-eGFP^C-tag^-Bba_B0015 | Dual C-terminal eGFP fusion tag and Bba_B0015 terminator | BsaI (PCR) |
| pSJM893 | pBP-ORF-*sfGFP* | Synthetic gene codon optimised for expression in *Streptomyces* spp. | BsaI (PCR) |
| pSJM899 | pBP-*kasOp** | *Streptomyces* constitutive promoter (active in *E. coli*) | Oligo anneal |

**Table S4.** Oligonucleotides used in this study

| **Oligo ID** | **Sequence (5’-3’)** |
| --- | --- |
| **Degenerate promoter library primers** | |
| Sigma70_11N_R | GCTTCTAGAACGTCTCAATCTGGTCTCACTATTTKAYRGCNNNNNNAGTCCTAGGKAYWGTNNNNNCGTACAGAGACCGGATCCCAC |
| Sigma70_F | CGCAGATCTTTTAACTTTAAGAAGGAGATATACATATGC |
| **PCR sub-cloning primers** | |
| Kan^R^_NdeI | CACCATATGAGATTGCAGCATTACACG |
| Kan^R^_BamHI | CACGGATCCGTCGACCTGCAGTTCGAAG |
| GFP-C-tag_F | CACGGTCTCATCGAGCCGTAAAGGAGAAGAACTTTTC |
| GFP-C-tag_R | GTGGGTCTCTAACATATAAACGCAGAAAGG |
| TAL_NdeI_NS | CACCATATGGCACCGCGTCCGACCAG |
| TAL_BamHI_NS | GTGGGATCCGGCCAGCATTTTCAGCAGAAC |
| PCL_NdeI_NS | CACCATATGGCACCGCAAGAACAGGC |
| PCL_BamHI_NS | GTGGGATCCCAGACCATTTGCCAGTTTGG |
| BAS_NdeI_NS | CACCATATGGCAACCGAAGAGATGAAAAAACTGG |
| BAS_BamHI_NS | GTGGGATCCGCTAATAACCGGAACGCTACG |
| **Oligo anneals** | |
| kasOp_F | TAGGTCTCACTATTTGACAACATGCTGTGCGGTGTTAAAGTGTACAGAGACCCATG |
| kasOp_R | GGTCTCTGTACACTTTAACACCGCACAGCATGTTGTCAAATAGTGAGACC |
| **Sequencing primers** | |
| TB | GGGTGTTAAAGCACGTCGTGG |
| PB | GATAGCGAACTGAGCGAAGATGATG |

Synthetic genes used in this study

*Tyrosine Ammonia Lyase (Rhodotorula glutinis)*

CAT**ATG**GCACCGCGTCCGACCAGCCAGAGCCAGGCACGTACCTGTCCGACCACACAGGTTACCCAGGTTGATATTGTTGAAAAAATGCTGGCAGCACCGACCGATAGCACCCTGGAACTGGATGGTTATAGCCTGAATCTGGGTGATGTTGTTAGCGCAGCACGTAAAGGTCGTCCGGTTCGTGTTAAAGATAGTGATGAAATTCGCAGCAAAATCGATAAAAGCGTGGAATTTCTGCGTAGCCAGCTGAGCATGAGCGTTTATGGTGTTACCACCGGTTTTGGTGGTAGCGCAGATACCCGTACCGAAGATGCAATTAGCCTGCAGAAAGCACTGCTGGAACATCAGCTGTGTGGTGTTCTGCCGAGCAGCTTTGATAGCTTTCGTCTGGGTCGTGGTCTGGAAAATAGCCTGCCGCTGGAAGTTGTTCGTGGTGCAATGACCATTCGTGTGAATAGCCTGACCCGTGGTCATAGCGCAGTTCGTCTGGTTGTTCTGGAAGCACTGACCAATTTTCTGAATCATGGTATTACCCCGATTGTTCCGCTGCGTGGCACCATTAGCGCAAGCGGTGATCTGAGTCCGCTGAGCTATATTGCAGCAGCAATTAGCGGTCATCCGGATAGCAAAGTTCATGTTGTTCATGAGGGCAAAGAGAAAATTCTGTACGCACGTGAAGCAATGGCACTGTTTAATCTGGAACCGGTTGTGCTGGGTCCGAAAGAAGGTCTGGGCCTGGTTAATGGTACAGCAGTTAGCGCCAGCATGGCAACCCTGGCACTGCATGATGCACACATGCTGAGCCTGCTGAGTCAGAGCCTGACCGCAATGACCGTTGAAGCCATGGTTGGTCATGCAGGTAGCTTTCATCCGTTTCTGCACGATGTTACCCGTCCGCATCCGACCCAGATTGAAGTTGCAGGTAATATTCGTAAACTGCTGGAAGGTAGCCGTTTTGCAGTTCATCATGAAGAAGAGGTGAAAGTCAAAGATGATGAAGGTATTCTGCGTCAGGATCGTTATCCGCTGCGCACCAGTCCGCAGTGGCTGGGTCCTCTGGTTAGCGATCTGATTCATGCACATGCAGTTCTGACCATTGAAGCAGGTCAGAGTACCACCGATAATCCGCTGATTGATGTTGAAAACAAAACCAGCCATCATGGTGGTAATTTTCAGGCAGCAGCAGTTGCAAATACCATGGAAAAAACACGCCTGGGTCTGGCACAGATTGGTAAACTGAATTTTACCCAGCTGACCGAAATGCTGAATGCAGGTATGAATCGTGGCCTGCCGAGCTGTCTGGCAGCAGAAGATCCGAGCCTGAGTTATCATTGTAAAGGTCTGGATATTGCCGCAGCCGCATATACCAGCGAACTGGGTCATCTGGCAAATCCGGTTACCACCCATGTTCAGCCTGCCGAAATGGCAAATCAGGCAGTTAATAGCCTGGCCCTGATTAGCGCACGTCGCACCACCGAAAGCAATGATGTTCTGAGTCTGCTGCTGGCAACCCATCTGTATTGTGTGCTGCAGGCCATTGATCTGCGTGCAATTGAATTTGAGTTCAAAAAACAGTTTGGTCCGGCAATTGTTAGCCTGATTGATCAGCATTTTGGTAGCGCCATGACCGGTAGCAATCTGCGTGATGAGCTGGTTGAAAAAGTGAATAAAACCCTGGCCAAACGTCTGGAACAGACCAATAGCTATGATCTGGTTCCGCGTTGGCATGATGCCTTTAGCTTTGCAGCAGGCACCGTTGTTGAAGTTCTGAGCAGCACCAGCCTGTCACTGGCAGCCGTTAATGCATGGAAAGTTGCAGCGGCAGAAAGCGCAATTAGTCTGACCCGTCAGGTTCGTGAAACCTTTTGGAGCGCAGCAAGCACCAGCAGTCCGGCACTGAGCTATCTGTCACCGCGTACACAGATTCTGTATGCATTTGTTCGTGAAGAACTGGGTGTTAAAGCACGTCGTGGTGATGTTTTTCTGGGTAAACAAGAAGTTACCATTGGTAGCAATGTGAGCAAAATCTATGAAGCCATTAAAAGCGGTCGCATTAATAACGTTCTGCTGAAAATGCTGGCC**TAA**GGATCCTCGA

*p-coumarate CoA Ligase (Arabidopsis thaliana)*

CAT**ATG**GCACCGCAAGAACAGGCAGTTAGCCAGGTTATGGAAAAACAGAGCAACAATAACAACAGCGACGTGATTTTTCGTAGCAAACTGCCGGATATCTATATTCCGAATCATCTGAGCCTGCACGATTATATCTTTCAGAACATTAGCGAGTTTGCCACCAAACCGTGTCTGATTAATGGTCCGACCGGTCATGTTTATACCTATAGTGATGTTCATGTGATCAGCCGTCAGATTGCAGCCAATTTTCATAAACTGGGTGTGAATCAGAATGACGTTGTTATGCTGCTGCTGCCGAATTGTCCGGAATTTGTTCTGAGCTTTCTGGCAGCAAGCTTTCGTGGTGCAACCGCAACCGCAGCAAATCCGTTTTTTACACCGGCAGAAATTGCAAAACAGGCAAAAGCAAGCAACACCAAACTGATTATTACCGAAGCACGTTACGTGGACAAAATCAAACCGCTGCAGAATGATGATGGTGTTGTGATTGTTTGCATCGATGATAATGAAAGCGTTCCGATTCCGGAAGGTTGTCTGCGTTTTACCGAACTGACCCAGAGCACCACCGAAGCAAGCGAAGTTATTGATAGCGTTGAAATTAGTCCGGATGATGTTGTTGCACTGCCGTATAGCAGCGGCACCACCGGTCTGCCGAAAGGTGTGATGCTGACCCATAAAGGTCTGGTTACCAGCGTTGCACAGCAGGTTGATGGTGAAAATCCGAATCTGTATTTTCATAGCGACGATGTTATTCTGTGTGTGCTGCCGATGTTTCATATTTATGCACTGAATAGCATTATGCTGTGTGGTCTGCGTGTTGGTGCAGCAATTCTGATTATGCCGAAATTTGAAATTAACCTGCTGCTGGAACTGATTCAGCGTTGTAAAGTTACCGTTGCACCGATGGTTCCGCCTATTGTTCTGGCCATTGCAAAAAGCAGCGAAACCGAAAAATATGATCTGAGCAGCATTCGTGTTGTTAAAAGCGGTGCAGCTCCGCTGGGTAAAGAACTGGAAGATGCAGTTAATGCCAAATTTCCGAATGCAAAACTGGGTCAGGGTTATGGTATGACCGAAGCAGGTCCGGTTCTGGCAATGAGCCTGGGTTTTGCCAAAGAACCGTTTCCGGTTAAATCAGGTGCATGTGGCACCGTTGTTCGTAATGCAGAAATGAAAATCGTTGATCCGGATACCGGTGATAGCCTGAGCCGTAATCAGCCTGGTGAAATTTGTATTCGTGGTCACCAGATTATGAAAGGCTATCTGAATAATCCGGCAGCCACCGCAGAAACCATTGATAAAGATGGTTGGCTGCATACAGGTGATATTGGTCTGATTGATGATGATGACGAACTGTTTATTGTGGATCGTCTGAAAGAGCTGATCAAATACAAAGGTTTTCAGGTTGCTCCTGCAGAGCTGGAAGCACTGCTGATTGGTCATCCGGATATTACCGATGTTGCAGTTGTTGCAATGAAAGAAGAAGCAGCAGGCGAAGTTCCGGTTGCATTTGTTGTGAAAAGCAAAGATAGCGAACTGAGCGAAGATGATGTTAAACAGTTTGTTAGCAAACAGGTGGTGTTCTACAAACGCATCAACAAAGTGTTTTTTACCGAGAGCATTCCGAAAGCACCGAGCGGTAAAATTCTGCGTAAAGACCTGCGTGCCAAACTGGCAAATGGTCTG**TAA**GGATCCTCGA

*Benzalacetone Synthase (Rheum palmatum)* CAT**ATG**GCAACCGAAGAGATGAAAAAACTGGCAACCGTTATGGCAATTGGCACCGCAAATCCGCCTAATTGTTATTATCAGGCAGATTTCCCGGATTTCTATTTTCGTGTTACCAATAGCGATCACCTGATCAACCTGAAACAGAAATTCAAACGTCTGTGCGAAAACAGCCGTATCGAAAAACGTTATCTGCATGTGACCGAAGAAATCCTGAAAGAAAATCCGAATATCGCAGCCTATGAAGCAACCAGCCTGAATGTTCGTCATAAAATGCAGGTTAAAGGTGTTGCAGAACTGGGTAAAGAAGCAGCACTGAAAGCAATTAAAGAATGGGGTCAGCCGAAAAGCAAAATTACCCATCTGATTGTTTGTTGTCTGGCAGGCGTTGATATGCCTGGTGCAGATTATCAGCTGACCAAACTGCTGGATCTGGACCCGAGCGTTAAACGTTTTATGTTTTATCATCTGGGTTGTTATGCCGGTGGCACCGTTCTGCGTCTGGCAAAAGATATTGCAGAAAATAACAAAGGTGCCCGTGTTCTGATTGTGTGTAGCGAAATGACCACCACCTGTTTTCGTGGTCCGAGCGAAACCCATCTGGATAGCATGATTGGTCAGGCAATTCTGGGTGATGGTGCAGCAGCAGTTATTGTTGGTGCCGATCCTGATCTGACCGTTGAACGTCCGATTTTTGAACTGGTTAGCACCGCACAGACCATTGTTCCGGAAAGCCATGGTGCAATTGAAGGTCATCTGCTGGAAAGCGGTCTGAGCTTTCATCTGTATAAAACCGTTCCGACCCTGATTAGCAACAACATTAAAACCTGTCTGTCCGATGCATTTACACCGCTGAATATTAGCGATTGGAATAGCCTGTTTTGGATTGCACATCCGGGTGGTCCGGCTATTCTGGATCAGGTTACCGCAAAAGTTGGTCTGGAAAAAGAAAAACTGAAAGTTACCCGTCAGGTGCTGAAAGATTATGGTAATATGAGCAGCGCAACCGTGTTTTTTATCATGGATGAAATGCGCAAAAAAAGCCTGGAAAATGGTCAGGCCACCACCGGTGAAGGTCTGGAATGGGGTGTTCTGTTTGGTTTTGGTCCGGGTATTACCGTTGAAACCGTTGTTCTGCGTAGCGTTCCGGTTATTAGC**TAA**GGATCCTCGA

*Malonyl-CoA Synthetase (Rhodopseudomonas palustris)*

CAT**ATG**AACGCAAACCTGTTTGCACGCCTGTTTGATAAACTGGATGATCCGCATAAACTGGCAATTGAAACCGCAGCCGGTGATAAAATCAGCTATGCAGAACTGGTTGCACGTGCCGGTCGTGTTGCAAATGTTCTGGTTGCCCGTGGTCTGCAGGTTGGTGATCGTGTTGCCGCACAGACCGAAAAAAGCGTTGAAGCACTGGTTCTGTATCTGGCAACCGTTCGTGCGGGTGGTGTTTATCTGCCGCTGAATACCGCATATACCCTGCATGAACTGGATTATTTCATTACCGATGCCGAACCGAAAATTGTTGTTTGTGATCCGAGCAAACGTGATGGTATTGCAGCAATTGCAGCCAAAGTTGGTGCAACCGTTGAAACCCTGGGTCCGGATGGTCGTGGTAGCCTGACCGATGCAGCAGCGGGTGCAAGCGAAGCATTTGCCACCATTGATCGTGGTGCAGATGATCTGGCAGCAATTCTGTATACCAGCGGCACCACCGGTCGTAGCAAAGGTGCAATGCTGAGCCATGATAATCTGGCAAGTAATAGTCTGACCCTGGTTGATTATTGGCGTTTTACACCGGATGATGTTCTGATTCATGCACTGCCGATTTATCATACCCATGGTCTGTTTGTTGCAAGCAATGTTACCCTGTTTGCGCGTGGTAGCATGATTTTTCTGCCGAAATTTGATCCGGACAAAATCCTGGATCTGATGGCACGTGCGACCGTGCTGATGGGTGTTCCGACCTTTTATACACGTCTGCTGCAGAGTCCGCGTCTGACCAAAGAAACCACAGGTCACATGCGTCTGTTTATTAGCGGTTCAGCACCGCTGCTGGCAGATACCCATCGTGAATGGTCAGCAAAAACCGGTCATGCAGTTCTGGAACGTTATGGTATGACCGAAACCAATATGAATACCAGCAATCCGTATGATGGTGATCGCGTTCCGGGTGCAGTTGGTCCGGCACTGCCTGGTGTTAGCGCACGTGTTACCGATCCGGAAACCGGTAAAGAACTGCCTCGTGGTGATATTGGTATGATTGAAGTTAAAGGTCCGAACGTGTTTAAAGGCTATTGGCGTATGCCGGAAAAAACCAAAAGCGAATTTCGTGATGATGGCTTTTTTATCACAGGTGATCTGGGCAAAATTGATGAACGTGGTTATGTTCATATTCTGGGTCGCGGTAAAGATCTGGTTATTACCGGTGGTTTTAACGTGTATCCGAAAGAAATTGAAAGCGAGATTGATGCAATGCCAGGTGTTGTTGAAAGCGCAGTTATTGGTGTTCCTCATGCAGATTTTGGTGAAGGTGTTACCGCAGTTGTTGTGCGTGATAAAGGTGCGACCATTGATGAAGCCCAGGTTCTGCATGGTCTGGATGGTCAGCTGGCAAAATTCAAAATGCCTAAAAAAGTGATCTTCGTGGATGATCTGCCTCGTAATACCATGGGCAAAGTTCAGAAAAATGTGCTGCGTGAAACCTACAAAGACATCTATAAA**TGA**GGATCCTCGA

*Malonate transporter* (*Rhizobium trifolii*)

CAT**ATG**GGTATTGAACTGCTGAGCATTGGTCTGCTGATTGCCATGTTTATTATCGCAACCATTCAGCCGATTAATATGGGTGCACTGGCATTTGCCGGTGCATTTGTTCTGGGTAGCATGATTATTGGCATGAAAACCAACGAAATCTTTGCAGGTTTTCCGAGCGACCTGTTTCTGACCCTGGTTGCAGTTACCTACCTGTTTGCAATTGCCCAGATTAATGGCACCATTGATTGGCTGGTTGAATGTGCAGTTCGTCTGGTTCGTGGTCGTATTGGTCTGATTCCGTGGGTTATGTTTCTGGTTGCCGCAATTATTACCGGTTTTGGTGCCCTGGGTCCGGCAGCAGTTGCAATTCTGGCACCGGTTGCACTGAGCTTTGCAGTTCAGTATCGTATTCATCCGGTTATGATGGGTCTGATGGTTATTCATGGTGCACAGGCAGGCGGTTTTAGCCCGATTAGCATTTATGGTGGTATTACCAATCAGATTGTTGCAAAAGCAGGTCTGCCGTTTGCACCGACCAGTCTGTTTCTGTCAAGCTTTTTTTTCAATCTGGCCATTGCCGTGCTGGTGTTTTTTGTTTTTGGTGGTGCACGTGTGATGAAACATGATCCGGCAAGTCTGGGTCCGCTGCCGGAACTGCATCCGGAAGGTGTTAGCGCAAGCATTCGTGGTCATGGTGGCACTCCGGCAAAACCGATTCGTGAACATGCCTATGGCACCGCAGCAGATACCGCAACCACCCTGCGTCTGAATAATGAACGTATTACCACCCTGATTGGCCTGACCGCACTGGGTATTGGTGCGCTGGTTTTCAAATTTAACGTTGGTCTGGTTGCAATGACCGTTGCCGTTGTTCTGGCCCTGCTGAGCCCGAAAACCCAGAAAGCAGCAATTGATAAAGTTAGCTGGTCAACCGTTCTGCTGATCGCAGGTATTATTACCTATGTTGGTGTTATGGAAAAAGCAGGCACCGTTGATTATGTTGCAAATGGTATTAGCAGCCTGGGTATGCCGCTGCTGGTTGCCCTGCTGCTGTGTTTTACCGGTGCAATTGTGAGCGCATTTGCAAGCAGCACCGCACTGCTGGGTGCAATTATTCCGCTGGCAGTTCCGTTTCTGCTGCAGGGTCATATTAGCGCAATTGGTGTTGTTGCAGCCATTGCAATTAGCACCACCATTGTTGATACCAGCCCGTTTAGCACCAATGGCGCACTGGTTGTTGCCAACGCACCGGATGATAGCCGTGAACAGGTTCTGCGTCAGCTGCTGATTTATAGCGCACTGATTGCGATTATTGGTCCGATTGTTGCCTGGCTGGTTTTTGTTGTTCCGGGTCTGGTT**TAA**GGATCCTCGA

*Raspberry Ketone Synthase (Rubeus rubrum)*

CAT**ATG**GCAAGCGGTGGTGAAATGCAGGTTAGCAATAAACAGGTGATCTTTCGTGATTATGTTACCGGCTTTCCGAAAGAAAGCGATATGGAACTGACCACCCGTAGCATTACCCTGAAACTGCCGCAGGGTAGCACCGGTCTGCTGCTGAAAAATCTGTATCTGAGCTGTGATCCGTATATGCGTGCACGTATGACCAATCATCATCGTCTGAGCTATGTGGATAGCTTTAAACCGGGTAGCCCGATTATTGGTTATGGTGTTGCACGTGTTCTGGAAAGCGGTAATCCGAAATTCAATCCGGGTGATCTGGTTTGGGGTTTTACCGGTTGGGAAGAATATAGCGTTATTACCGCAACCGAAAGCCTGTTCAAAATTCATAATACCGATGTTCCGCTGAGCTATTATACAGGTCTGCTGGGTATGCCTGGTATGACCGCCTATGCAGGTTTTTATGAAATTTGCAGCCCGAAAAAAGGCGAAACCGTTTATGTTAGCGCAGCCAGCGGTGCAGTTGGTCAGCTGGTTGGTCAGTTCGCAAAACTGACCGGTTGTTATGTTGTTGGTAGCGCAGGTAGCAAAGAAAAAGTTGATCTGCTGAAAAACAAATTCGGCTTTGATGAGGCCTTCAACTATAAAGAAGAAGCAGATCTGGACGCAGCACTGCGTCGTTATTTTCCGGATGGTATTGATATCTATTTCGAAAACGTGGGTGGCAAAATGCTGGATGCAGTTCTGCCGAATATGCGTCCGAAAGGTCGTATTGCAGTTTGTGGTATGATTAGCCAGTATAATCTGGAACAGCCGGAAGGTGTTCGTAATCTGATGGCACTGATTGTTAAACAGGTTCGCATGGAAGGCTTTATGGTCTTTAGCTATTATCACCTGTACGGCAAATTTCTGGAAACCGTGCTGCCGTATATCAAACAGGGTAAAATTACCTATGTGGAAGATGTTGTTGATGGCCTGGATAATGCACCGGCAGCCCTGATTGGTCTGTATAGCGGTCGTAATGTTGGCAAACAGGTTGTTGTTGTTAGCCGTGAA**TAA**GGATCCTCGA

*sfGFP (Synthetic codon-optimised for Streptomyces spp.)*

CAT**ATG**TCCAAGGGCGAGGAGCTGTTCACCGGCGTCGTCCCGATCCTGGTCGAGCTGGACGGCGACGTGAACGGCCACAAGTTCTCCGTCCGCGGCGAGGGCGAGGGCGACGCCACCAACGGCAAGCTGACCCTGAAGTTCATCTGCACCACCGGCAAGCTCCCGGTCCCGTGGCCGACCCTGGTCACCACCCTGACCTACGGCGTCCAGTGCTTCTCCCGCTACCCGGACCACATGAAGCGCCACGACTTCTTCAAGTCCGCCATGCCCGAGGGCTACGTCCAGGAGCGGACCATCTCCTTCAAGGACGACGGCACCTACAAGACCCGCGCCGAGGTCAAGTTCGAGGGCGACACCCTGGTCAACCGCATCGAGCTGAAGGGCATCGACTTCAAGGAGGACGGCAACATCCTGGGCCACAAGCTCGAGTACAACTTCAACTCCCACAACGTCTACATCACCGCCGACAAGCAGAAGAACGGCATCAAGGCCAACTTCAAGATCCGCCACAACGTCGAGGACGGCAGCGTCCAGCTGGCCGACCACTACCAGCAGAACACCCCGATCGGCGACGGCCCGGTCCTGCTGCCGGACAACCACTACCTGTCCACCCAGTCCGTCCTGTCCAAGGACCCGAACGAGAAGCGCGACCACATGGTCCTGCTCGAGTTCGTCACCGCCGCCGGCATCACCCACGGCATGGACGAGCTGTACAAGTGAGGATCCTCGA

References

Guerrero Montero, I., Richards, K.L., Jawara, C., Browning, D.F., Peswani, A.R., Labrit, M., Allen, M., Aubry, C., Davé, E., Humphreys, D.P., Busby, S.J.W., Robinson, C., 2019. *Escherichia coli* “TatExpress” strains export several g/L human growth hormone to the periplasm by the Tat pathway. Biotechnol. Bioeng. 116, 3282–3291.
