# Supplementary material for: Refactoring of a synthetic raspberry ketone pathway with EcoFlex
Source: Microb Cell Fact. 2021 Jun 10;20:116. doi: 10.1186/s12934-021-01604-4 (PMC8193874; doi:10.1186/s12934-021-01604-4)
Supplement: Supplementary file 2 — Additional file 2. Extracted ion chromatograms and example LC-MS calibration data. [file 12934_2021_1604_MOESM2_ESM.docx]

**Additional File 2**

LC/MS extracted ion chromatogram corresponding to the [M+H]^+^ ion for L-Tyrosine (*m/z* - 182.081): Cultured supernatant (upper) and standard solution (lower)

LC/MS extracted ion chromatogram corresponding to the [M+H]^+^ ion for *p*-coumarate (*m/z* - 165.054): Cultured supernatant (upper) and standard solution (lower)

LC/MS extracted ion chromatogram corresponding to the [M+H]^+^ ion for HBA (*m/z* - 163.075): Cultured supernatant (upper) and standard solution (lower)

LC/MS extracted ion chromatogram corresponding to the sodium adduct [M+Na]^+^ of raspberry ketone (*m/z* - 187.073): Cultured supernatant (upper) and standard solution (lower).

Example LC-MS calibration standards for quantitation of raspberry ketone intermediates
